# Spatiotemporal Diversification of Global Junipers: Traces of Niche Conservatism and Trait‐Dependent Diversification

**DOI:** 10.1002/ece3.70910

**Published:** 2025-02-12

**Authors:** Rodrigo Martínez de León, Alejandra Moreno‐Letelier

**Affiliations:** ^1^ Posgrado en Ciencias Biológicas Universidad Nacional Autónoma de México Ciudad de México Mexico; ^2^ Jardín Botánico, Instituto de Biología Universidad Nacional Autónoma de México Ciudad de México Mexico

## Abstract

Speciation rates differ globally across phylogenies and regions, and the complexity of the speciation process makes it difficult to fully understand these variations. It has been suggested that in conifers, the speciation process is primarily influenced by abiotic factors, operating in a local adaptive dynamic. In this study, we investigate changes in the climatic envelopes of conifer species of the genus *Juniperus* and test their explanatory power in predicting the distribution of speciation rates in space controlling for other explanatory factors such as topography and morphology. Location: Northern Hemisphere. Taxon: *Juniperus.* We used phylogenetic comparative methods and macroecological methods to evaluate speciation rate shifts, trait‐speciation correlations, trait‐geography correlations, and areas of phylogenetic endemism. Climatic niches in the juniper species follow bimodal trends in temperature and precipitation variables. These trends imply a strong event of divergence or possible adaptation that is trackable to a specific moment and geography. Additionally, we found significant explanatory power for some climatic variables and a heterogeneous response by continent, with morphological changes being the most important in North America, whereas climate is more relevant in the Tibetan Plateau. Centers of diversity follow different trends in phylogenetic diversity and endemism, suggesting different causes of diversity. Overall, junipers exhibit a conserved climatic niche, and their speciation process is marked by the particular history of their distribution rather than by general trends in climatic evolution. Morphological evolution is more important than previously considered and both accumulation of lineages and rapid speciation are supported for hotspot regions.

## Graphical abstract

Conifer diversification does not follow a single cause. The diversification of Junipers in the Tibetan Plateau responded to climate, but in North America, it was caused by morphological evolution.

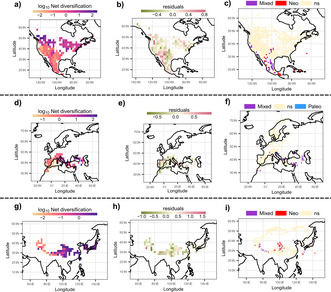

## 1 Introduction

Plant speciation, the process by which plant lineages split over time, is a fluctuating process rather than a constant across the tree of life. Some lineages have higher rates of diversification than others, even among genera within the same family (Hernández‐Hernández et al. 2021; Tank et al. 2015). Such disparities in the speciation–extinction equilibrium (i.e., the diversification process) result in heterogeneity of plant diversity worldwide (Donoghue and Sanderson 2015). Plant diversity hotspots represent only 15.7% of the land (not counting for habitat loss) and hold no less than 50% of the total plant species in existence (Marchese 2015; Mittermeier et al. 2011). The origin and distribution of such heterogeneous diversity remain a central question in plant biogeography, ecology, and systematics (Jablonski, Roy, and Valentine 2006; Soria‐Carrasco and Castresana 2012).

The current distribution of diversity is governed not only by the equilibrium in extinction and speciation processes, but also by the impacts of lineage migration, distributional shifts, and expansions (Jablonski, Roy, and Valentine 2006; Wiens and Donoghue 2004). For example, many lines of evidence suggest the existence of diversity hotspots in historically climatically stable regions when attempting to explain latitudinal diversity gradients (Harrison and Noss 2017; Sandel et al. 2011). Other lines of evidence highlight the importance of mountains in the maintenance and origin of biodiversity, either by acting as cradles or museums (Perrigo, Hoorn, and Antonelli 2020; Rahbek et al. 2019). The relationship between diversification heterogeneity and the factors that drive speciation and shape distribution cannot be overlooked in any discussion of these topics, whether those factors are intrinsic or extrinsic.

The various mechanisms through which the climatic component of ecological niches facilitates or impedes speciation have been extensively discussed in previous research (Boucher et al. 2014; Hua and Wiens 2013; Nosil, Harmon, and Seehausen 2009; Wiens 2004). The tendency of lineages to retain certain components of their ecological niche during evolutionary time is referred to as Phylogenetic Niche Conservatism (PNC) (Eaton, Soberón, and Peterson 2008; Pyron et al. 2015). The climatically guided speciation process represents a complex scenario in which geological history, species niche widths, and evolutionary forces take part. This process generates different patterns where speciation is facilitated either by labile climatic niches and heterogeneous environments or even in the presence of strong PNC in combination with strong geographic barriers (e.g., Gómez‐Rodríguez, Baselga, and Wiens 2015; Hua and Wiens 2013; Pyron et al. 2015; Warren et al. 2014; Wiens 2004). The questions regarding this process are if climatic specialization (i.e., niche divergence) is the main diversification driver in each lineage and whether a correlation exists between climatic factors and species diversity and diversification (Donoghue and Sanderson 2015; Gómez‐Rodríguez, Baselga, and Wiens 2015). A plethora of methods are available for the study of the evolution of climatic niches, including identity tests, phylogenetic‐niche similarity correlations, and model‐based approaches (Peixoto, Villalobos, and Cianciaruso 2017; Warren, Glor, and Turelli 2008; Warren et al. 2014). However, macroecology offers opportunities to integrate more complex hypotheses regarding the relationship between climatic evolution, diversification rates, and diversity altogether (Kling et al. 2019; Puglielli and Pärtel 2023).

Conifers are an economically important and evolutionary intriguing group of plants. They first appeared ~300 million years ago and have thrived and even are dominant, particularly in the Northern Hemisphere (Díaz‐Sala et al. 2013; Farjon 2003; Leslie et al. 2018). While the major conifer families date back to the Triassic Period (~252 to 201 mya), many conifer lineages are represented by only one species indicating that they might have gone through extensive extinction (Farjon 2003; Bolte and Eckert 2020). Notably, most of the diversity is concentrated in a few genera, especially *Pinus*, *Podocarpus*, and *Juniperus* (Eckenwalder 2009; Farjon 2010). Such diversity is relatively recent with genera like *Pinus*, *Abies*, and *Juniperus* having increased diversification during the Miocene (Aguirre‐Planter et al. 2012; Leslie et al. 2012, 2018; Uckele et al. 2021). Nevertheless, evidence suggests the existence of neutral processes governing the diversification of conifers, especially in regions where high phylogenetic endemism is found. Such processes include the accumulation of diversity and niche conservatism (Cruz‐Nicolás et al. 2023; Cruz‐Nicolás, Jaramillo‐Correa, and Gernandt 2024; Sundaram et al. 2019; Sundaram and Leslie 2021).

*Juniperus* L. is composed of 70–76 species and is the most diverse genus in the Cupressaceae family (Adams 2014; Farjon 2005). The genus is composed mostly of trees and shrubs, with berry‐like cones that do not open, and are dispersed by birds and small mammals when mature (Farjon 2005; Liu et al. 2022; Mao et al. 2019). According to Adams (2014), *Juniperus* has three main diversity hotspots globally: North America and the Caribbean, the Mediterranean Basin, and the Qinghai–Tibet Plateau (QTP) in central China. The biogeography of this conifer group implies major continental disjunctions and recurrent incursions into highly contrasting climatic conditions (e.g., the Caribbean islands or the Qinghai‐Plateau) which could indicate a possible adaptation to new climatic conditions (Adams 2014; Mao et al. 2010). Junipers represent a special case in conifer speciation research; unlike most conifers, juniper seeds are dispersed by animals, which is a key innovation that enhanced diversification and colonization to new habitats (Liu et al. 2024); moreover, they present a wide variety of life habits (e.g., trees, shrubs, and even prostrate shrubs) that may be key for their high diversity (Adams 2014).

In this study, we investigated the patterns of climatic niche evolution throughout the evolutionary history of juniper species and their impacts on diversification rates. We combined geospatial and phylogenetic information to test a niche‐related evolutionary scenario. Initially, we analyzed the extent of niche conservatism within the Juniper phylogeny using comparative phylogenetic methods. Second, we evaluated the relevance of morphological and niche characteristics on diversification rates, detected significant areas for the diversification of junipers, and proposed different causes for each. We expect that under a scenario of climate niche‐guided evolution, we would find strong supported shifts across the phylogeny which may overlap with the major distribution shifts reported. Additionally, we expected a high influence on climate variability, elevation, and morphological evolution on diversification rates.

## 2 Materials and Methods

### 2.1 Data Collection

To characterize the climatic niche of extant juniper species, we gathered geographic information from the GBIF.org database (25 April 2024. Download: https://doi.org/10.15468/dl.kej375) and filtered for 48 of the juniper species present in the phylogeny of Mao et al. (2010). Records were further cleaned and filtered with Coordinate Cleaner (Zizka et al. 2019) to discard records closer than 5 km within each other, records in herbaria, and records from cultivated individuals outside their distribution range (Table S1.1). We obtained the corresponding values of the final 13,446 records for the 19 bioclimatic layers available in CHELSA (at 30 arc resolution) (Karger et al. 2017). After reducing multicollinearity by filtering highly correlated variables (Pearson's *r* > |0.8|), we retained the following: BIO2 (mean diurnal air temperature range), BIO3 (isothermality), BIO6 (mean daily minimum air temperature of the coldest month), BIO7 (annual range of air temperature), BIO8 (mean daily mean air temperature of the wettest quarter), BIO9 (mean daily mean air temperature of the driest quarter), BIO10 (mean daily mean air temperature of the warmest quarter), BIO15 (precipitation seasonality), BIO16 (mean monthly precipitation of the wettest quarter), BIO17 (mean monthly precipitation of the driest quarter), BIO18 (mean monthly precipitation of the warmest quarter), and BIO19 (mean monthly precipitation of the coldest quarter) (Correlation matrix available on Table S2.1).

We reconstructed a phylogeny of *Juniperus* by retrieving all sequences used by Mao et al. (2010) (nine cDNA markers: *matK*, *rbcL*, *trnL‐F*, *rps4*, *trnS‐G*, *trnD‐T*, *trnV*, *petB‐D*, and *psbB*_*1*_ *‐B*_*2*_) and aligned them using MAFFT (Katoh and Standley 2013). We also used available sequences for *Cupressus* species and Clade CHX (*Callitropsis*, *Hesperocyparis*, and *Xanthocyparis*) for their use as outgroup. A concatenated alignment of all sequences was then used to build a maximum‐likelihood tree using IQ‐TREE (Nguyen et al. 2015). We used the GTR substitution model as reported by Mao et al. (2010) and obtained branch support with an UltraFast Bootstrap using 10,000 generations (Thi Hoang et al. 2017). We calibrated the resulting best tree using the penalized likelihood approach with the *chronos* function in the APE package (Kim and Sanderson 2008; Paradis, Claude, and Strimmer 2004) In the *chronos* function, we used the maximum and minimum clade ages estimated by Mao et al. (2010) as *secondary calibration* points, and a *λ* smoothing parameter of 1. We evaluated concordance with the results of Mao et al. (2010) and the recovery of the main well‐supported clades in the *Juniperus* tree in the original study, as well as divergence times (Figure S1.1).

### 2.2 Climatic Niche Evolution

We used the mean species values of each climatic variable as proxies for the climatic niche of each species and evaluated: (1) the evolutionary dynamics of the climatic niche of juniper species (i.e., the evolutionary trends) and (2) the rate of instant change in the evolution of these climatic variables (i.e., the velocity of change). For these purposes, we fitted two Bayesian algorithms in the phylogenetic tree and first used Bayou (Uyeda and Harmon 2014) to evaluate the existence of more than one climatic regime optimum (*θ*) in juniper climatic niche evolution under the assumption of a multi‐regime Ornstein–Uhlenbeck model. Second, we investigated shifts in the rates of climatic evolution over time using the BAMM software (Rabosky 2014). We used each climatic variable independently as the use of multivariate statistics (e.g., principal components and outlying mean indices) can be misleading (Uyeda, Caetano, and Pennell 2015).

We ran Bayou for 20 million iterations for each variable separately, discarding the first 10% as burn‐in. This multi‐regime OU approach models the number of adaptive regimes for each variable across phylogenies. Each adaptive (*k*_*i*_) regime is characterized by an optimum adaptive value *θ*, inertia or strength of selection around that value *α*, and rate of evolution *σ* ^2^ (Uyeda and Harmon 2014). We used *half‐Cauchy* prior distributions for *σ* ^2^ and *α*, and a conditional Poisson distribution with *λ* = 10 for the number of expected shifts in *θ* along the phylogeny. Convergence was assessed using CODA (Plummer et al. 2006) and the effective sample sizes were checked (ESS > 200; Table S1.2). Finally, we conserved the average map with at least one plausible shift event, with a posterior probability > 0.80.

We estimated the most suitable number of shifts in climatic niche evolution (i.e., changes in climatic niche rate) using the time‐dependent trait‐diversification model BAMM (Rabosky 2014). For each of the climatic variables, we performed two independent MCMC runs of 10 million generations using pre‐fitted priors with the function *setBAMMpriors* from the BAMMTools (Rabosky et al. 2014) package. MCMC were sampled every 10,000 generations and 10% were discarded as *burn‐in*. Convergence was verified and the most suitable number of major shifts in climatic niche diversification was obtained from the posterior probability distribution. Additionally, from this analysis, we retained the calculated tip metrics for each lineage of net diversification, speciation rate, and extinction rate for further analyses.

### 2.3 Geography and Diversification

We divided the world into a 2° × 2° grid and constructed an absence‐presence matrix with a total of 838 cells using the total 13,446 geographic records for the 48 *Juniperus* species with reliable geographic information.

From the BAMM runs, we extracted the diversification rates for each species (tip) and averaged it across every species cooccurring in each cell. Additionally, we calculated five different types of variables: diversity, morphological, physiographic, climate, and niche‐evolution for each cell, by averaging the tip metrics for the species cooccurring for each cell. *Diversity*: We first calculated the species richness, phylogenetic diversity (Faith 1992), and phylogenetic endemism (Rosauer et al. 2009) for each cell. *Morphological*: We also analyzed the rate of morphological evolution by examining the rate of transitions in two important characters of juniper species: the maximum number of seeds (0: multi‐seeded and 1: one‐seeded) and habit (0: tree form and 1: shrub form). Morphological data for each species were obtained from taxonomical descriptions in Adams (2014). We used the Herodotools R package (Nakamura et al. 2023) for stochastic mapping of ancestral states and the subsequent calculation of the transition rate, time of stasis, and last transition time for each cell and each character. *Physiographic*: We approximated the physiographic nature or regions by calculating the mean and standard deviation for the elevation of each cell. *Niche evolution*: We used the BAMM‐calculated *β* values (i.e., rates of trait evolution) from the niche evolution analysis described above for each species (tips) and averaged them across cells and lastly *Climate*: We used the average value for each cell for the 19 bioclimatic variables.

### 2.4 Statistical Analyses

We divided the data into three continental databases: North America, Eastern Asia, and Europe. To examine the connections between geographic variation in recent speciation rates and the five types of variables, we performed multiple linear regression analyses. Prior to the regression analysis, we made the effect size comparable among the predictors by standardizing and scaling the response and predictor variables (mean = 0; SD = 1). Additionally, we assessed multicollinearity among predictors by examining the variance inflation factor (VIF) (Dormann et al. 2013) and eliminating variables with higher values. This procedure was performed multiple times until the remaining variables had a VIF no greater than 6. We then calculated a general model using the R package MuMin (Barton and Barton 2015) and selected the best model for each continent based on the AIC criteria.

To quantify the relative contribution of each predictor in explaining the total variance in speciation rates, we performed hierarchical partitioning as implemented in the R package *relaimpo* (Grömping 2006). This procedure decomposes the model‐explained variance into non‐negative contributions and evaluates the relative importance of each predictor variable in the linear models (Grömping 2006). We further checked for spatial autocorrelation in the residuals of the selected models. We computed Moran's *I* based on residual speciation rates (i.e., the residuals from a linear multiple regression of predictors of speciation rates). We found low spatial autocorrelation in the residuals from the non‐spatial model for two regions (Eastern Asia and Europe). For the North American model, we used the same set of predictors to construct a spatial autoregressive error model *SARer* that considers the existence of spatial autocorrelation only in the residual distribution. Later, a Hausman test was performed to test if differences in the standardized coefficients of the non‐spatial and spatial models were significant.

### 2.5 Paleo‐ and Neo‐Endemism

To identify the geographical traces of juniper diversification, we conducted a categorical analysis of neo‐ and paleo‐endemism (CANAPE) (Mishler et al. 2014) using *Biodiverse* software (4.0) (Laffan, Lubarsky, and Rosauer 2010). Using the phylogenetic tree obtained above and the occurrence data, we assessed measures of phylogenetic diversity and phylogenetic endemism on a global scale using a 1° × 1° grid and determined the significance by 1000 randomizations. CANAPE seeks to identify regions in the distribution of species where significantly high phylogenetic endemism exists by comparing both the phylogenetic information and the distribution of species with scenarios produced by an equal‐length tree. Furthermore, it allows for the differentiation of areas dominated by rare long branches (paleo‐endemism) from those dominated by rare short branches (neo‐endemism) and rare branches of variable length (mixed endemism).

## 3 Results

### 3.1 Niche Evolution

**FIGURE 1:**
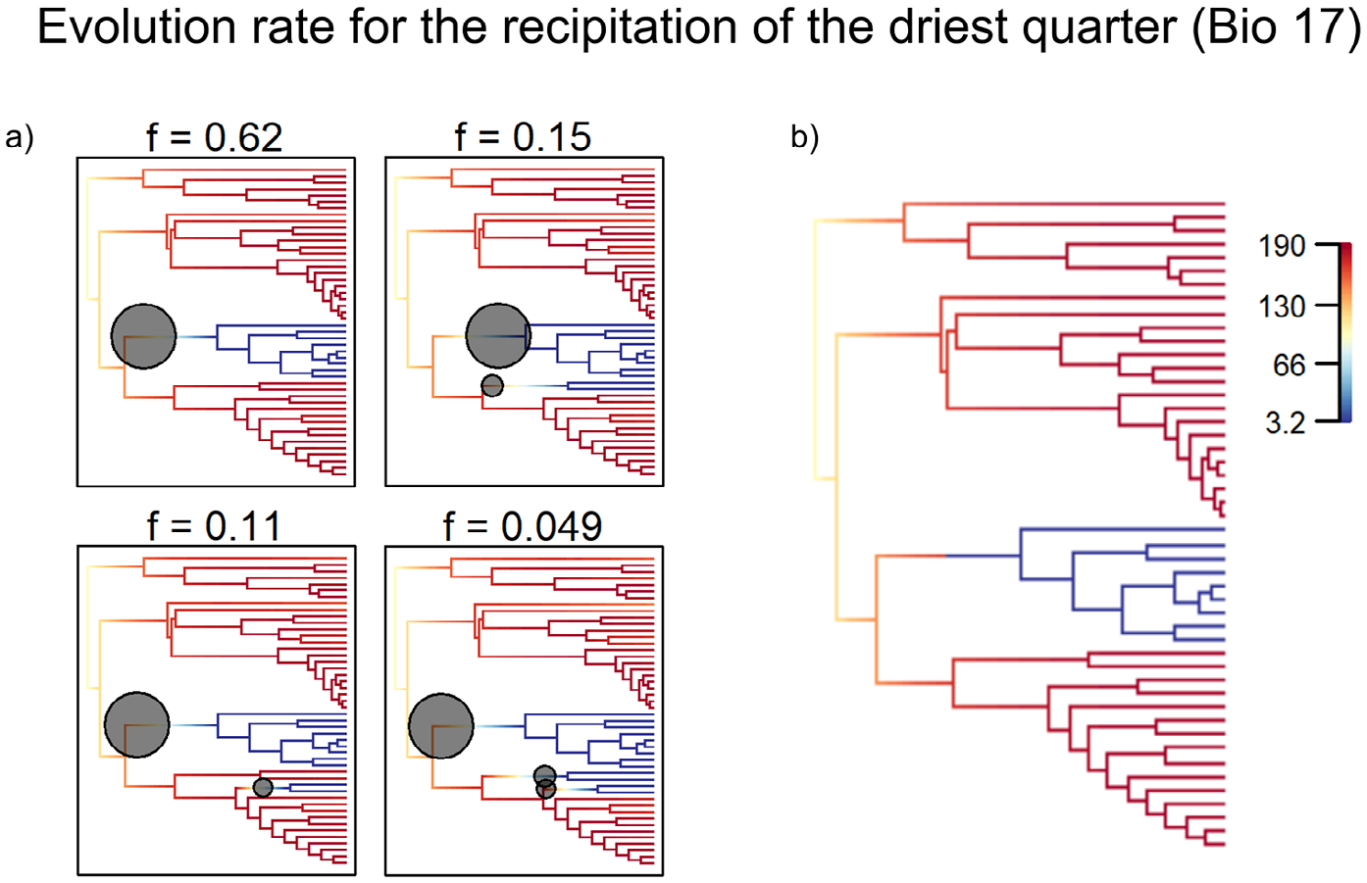
Credible shift configurations. (a) Set of configurations representing a marginal posterior probability > 0.8 for at least one rate shift in the Tibetan juniper clade and the frequency (*f*) of each scenario. Dark circles represent points in the character evolution where abrupt rate changes are estimated sized by their frequency on the posterior. (b) Best average configuration. Branch colors represent the instant rate of change for the Bio 17 variable as a phenotypic character.

For the temporal dynamics of climatic niche evolution, BAMM analysis showed significant heterogeneity in the trait rates of only one climatic variable (Bio17; Figure 1). According to our result, the Tibetan juniper clade of Section Sabina suffered an abrupt deceleration (marginal posterior probability > 0.8) in the evolution of tolerances to Bio 17(Precipitation of the driest quarter).

**FIGURE 2:**
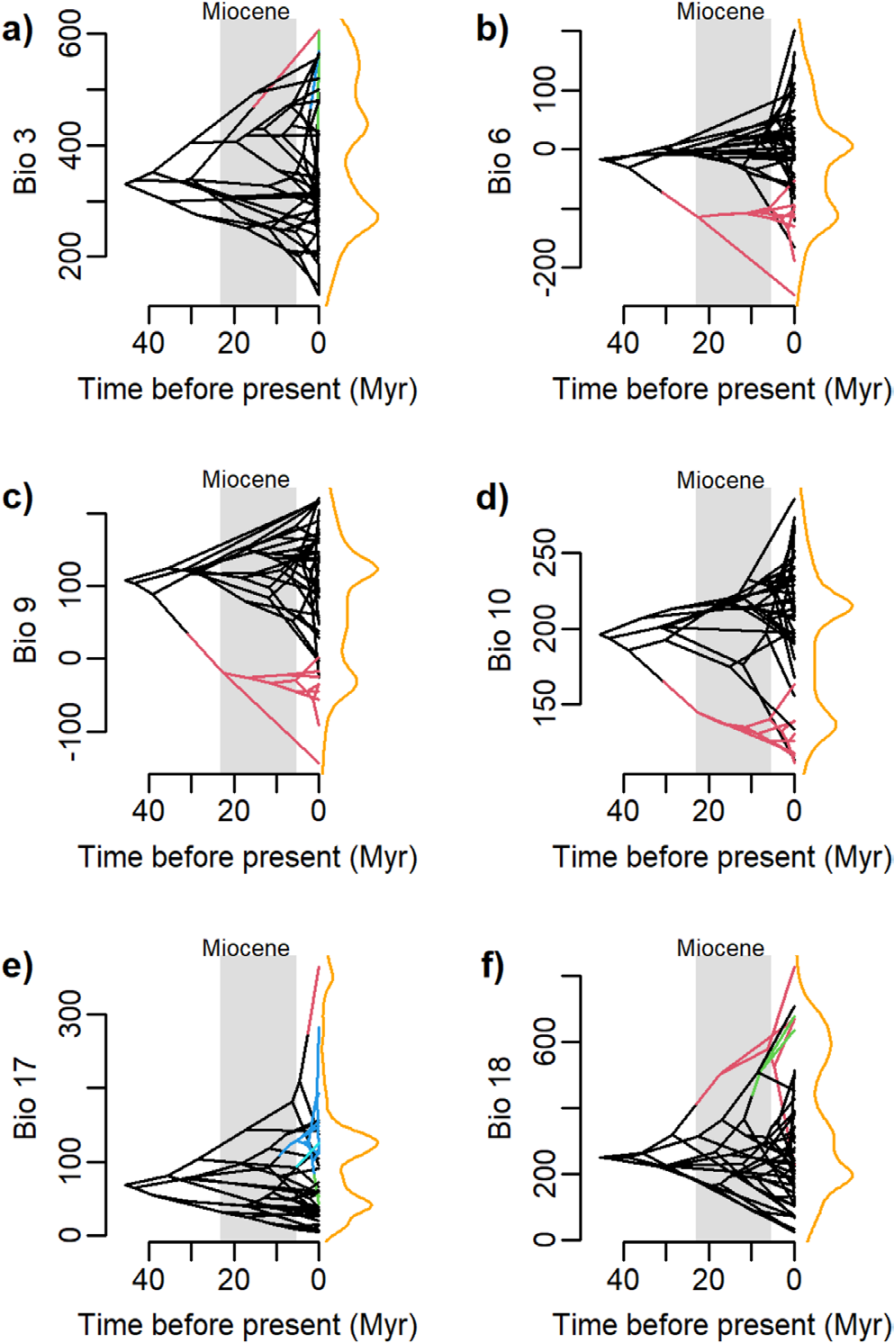
Evolutionary patterns of climatic optima evolution. Traitgram of the calculated regime evolution under 20 million Bayou iterations. Each optimum with posterior probability > 0.80 is represented by a different branch color. Density distribution of characters is represented by orange lines on the sides of each Traitgram and the Miocene (~23–5 mya) period is marked in the gray box. (a) Isothermality (Bio 3). (b) Mean minimum temperature of the coldest month (Bio 6). (c) Mean temperature of the driest quarter (Bio 9). (d) Mean temperature of the warmest month (Bio 10). (e) Precipitation of the driest quarter (Bio17). (f) Precipitation of the warmest quarter (Bio18).

Six bioclimatic variables showed significant regime shifts (posterior probability > 0.8) in the regime evolution analysis (i.e., Bayou analysis) (Figure 2): Bio3, Bio6, Bio9, Bio10, Bio17, and Bio18. Our Bayou results distinguished a clear regime shift in the temperature‐associated variables (Bio6, Bio9, and Bio10) for the Tibetan Plateau clade of junipers (Figure 2). Figure 2 also shows the general tendency of Tibetan junipers to grow at lower temperatures (marked in red). The variables associated with precipitation also showed significant regime shifts (Bio17 and Bio18) (Figure 2e,f); However, these regime shifts were found on singular species branches as well as the Caribbean clade of junipers (e.g., *Juniperus bermudiana*). Similarly, the analysis of isothermality also showed regime shifts in individual branches (Figure 2a) of juniper species in the three regions.

### 3.2 Geography and Diversification

**FIGURE 3:**
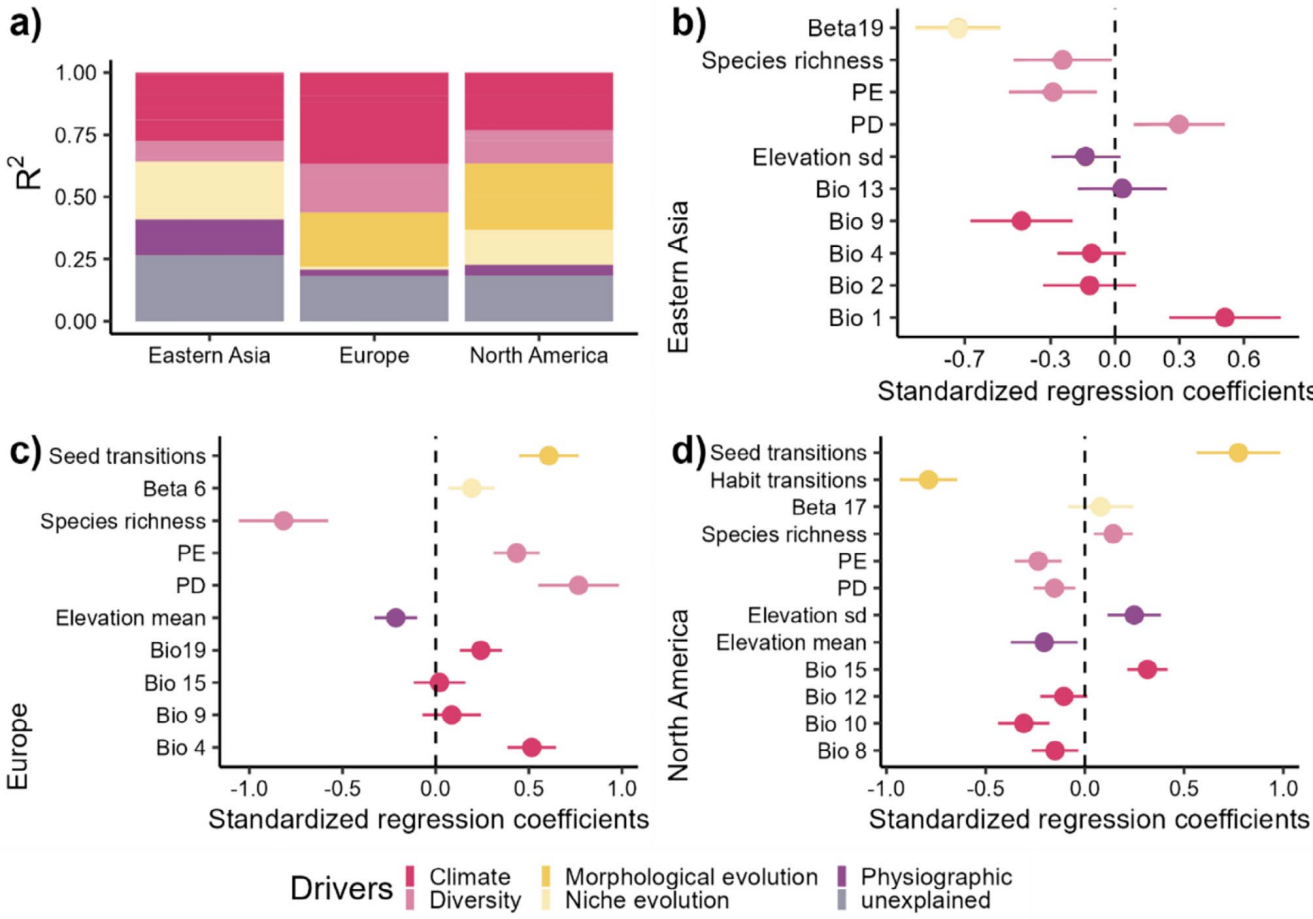
Predictors and their contribution to final models. (a) Variable's total explanatory contribution by their general classification and region. Each bar corresponds to one region and the total contribution of each type of variable to the final model's *R* ^2^. (b) Standardized regression coefficients and standard error for eastern Asia. (c) Standardized regression coefficients and standard error for Europe. (d) Standardized regression coefficients and standard error for North America.

The relative importance of each variable was calculated for the three different models to compare climatic and other plausible variables related to diversification. Details in regression coefficients and significance measures are available on Tables S3.1–S3.5. From this analysis, we found that climatic variables only hold for < 40% of total variation (Figure 3). The amount of variation in diversification rates explained by bioclimatic variables varied between regions (North America: 20.10%; Eastern Asia: 16.30% and Europe: 34.60%) with the European region having the most variance explained by bioclimatic variables and the Eastern Asian region having the least variance explained (Figure 3a).

In addition, we found a high relative importance for the variables used to reflect morphological evolution. Only the seed number transition rate holds for 43.74% of the total variation of the diversification rates in the North American region (Figure 3d). Meanwhile, 20.31% of the total variation in diversification rates for the European region can be explained by the variation in habit transition rates alone (Figure 3b,c).

Our final multiple regression models explained more than 80% of the total variance in diversification rates for the three regions (*R* ^2^ = 0.9148, *p* < 0.00001 for North and Central America; *R* ^2^ = 0.8272, *p* < 0.00001 for Easter Asia; and *R* ^2^ = 0.8823, *p* < 0.00001 for Europe). We found no signal of spatial autocorrelation in the residuals of the final models except the one for North America (Moran's *I*: 3.9703, *p* < 0.00001), nevertheless, we found no significant differences in the standardized coefficients between the non‐spatial and the spatial model for this region, as shown by the spatial Hausman test (9.1097, *p* = 0.6118). For this reason, we kept the original non‐spatial model standardized coefficients for further analyses. More information on partial residuals, individual variable contribution, and residuals vs. fitted values' plots are available in Sections S4–S6.

### 3.3 Phylogenetic Endemism

**FIGURE 4:**
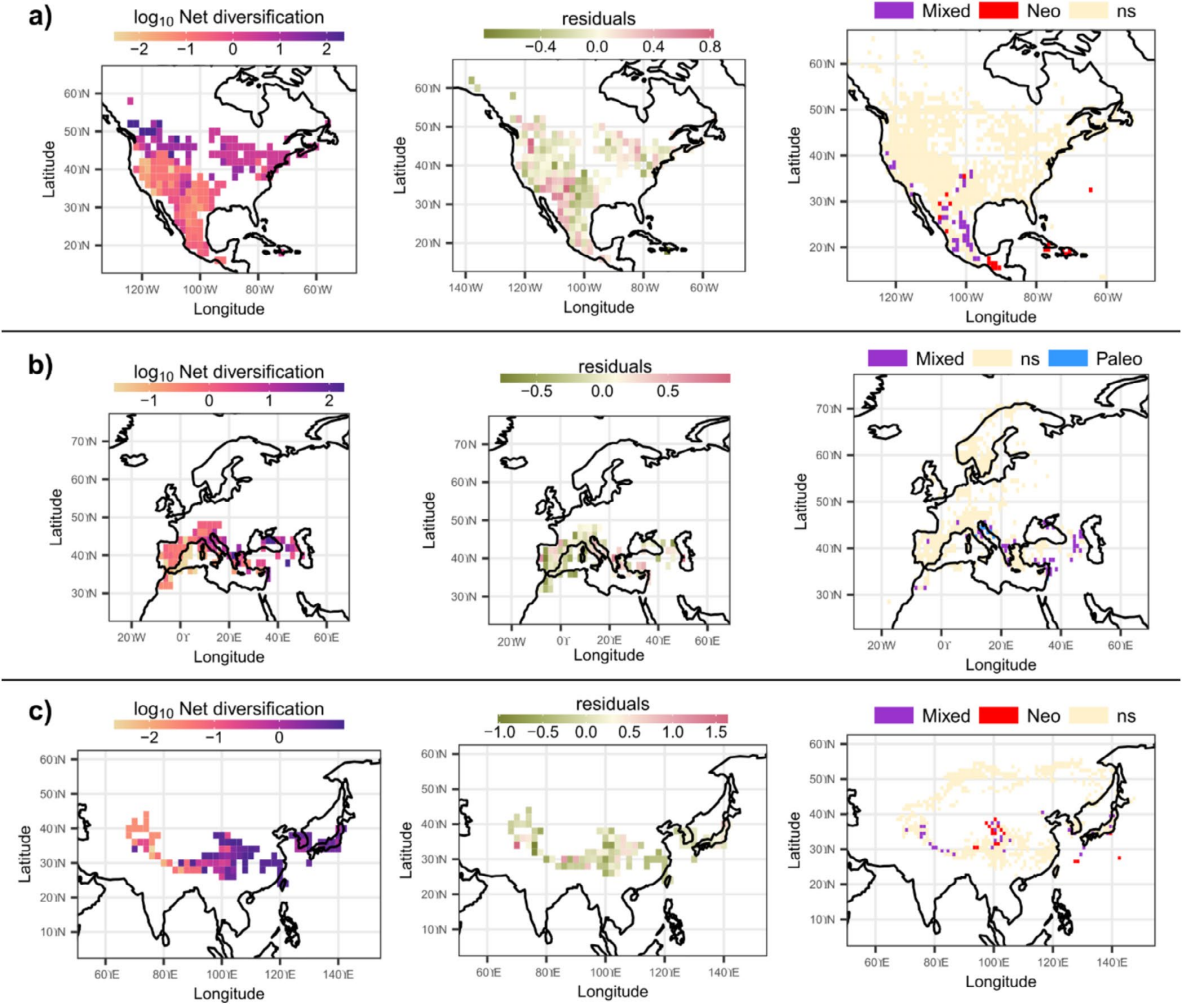
Patterns of diversification distribution and CANAPE results for each region. Distribution of net diversification index (left). Model residual's distribution (center) and CANAPE results (right) are shown for each region (row). (a) North and Central America, (b) Europe, and (c) Eastern Asia.

Our results on juniper phylogenetic diversity and endemism revealed the existence of four major diversification regions worldwide: the North Mesoamerica and the Antillean region (Figure 4a), Mediterranean Basin (Figure 4b), and the eastern Asian region along with the QTP (Figure 4c). While the four regions altogether showed a high prevalence of mixed endemism and no significant ultra‐endemic cells, significant neo‐endemic areas were found along the south and west of the Mexican highlands and the entire Antillean subregion (Figure 4a). For the European region, we found a prevalence of mixed endemism regions along the eastern part of the Mediterranean coast, with few cells containing significant paleo‐endemic regions (Figure 4b). The QTP region is another well‐supported region with a high prevalence of neo‐endemisms in our results, along with some pixels in eastern Asia (Figure 4c).

## 4 Discussion

We described the evolution of different climatic components of the climatic niches of juniper species and tested whether a group of candidate variables could explain the seen distribution of diversification rates at a global scale. We found that the distribution of diversification rates is related to different variables according to the continental area, suggesting that each of the three main centers of diversity has independent causes and origins. These results seem to be confirmed by the analyses on endemism, where different regions lead to very contrasting observations on endemism patterns. Our results on the climatic niche evolution of juniper lineages suggest that the history and diversification of this conifer genus are characterized by certain stability in climatic components of niches, which suggest an overall pattern of phylogenetic niche conservatism and vicariance‐guided speciation.

### 4.1 The Miocene Conundrum of Juniper Diversification

The Miocene period (~23 to 5 million years ago) is often recognized as significant for the diversification of conifer lineages, including junipers, pines, and firs (Aguirre‐Planter et al. 2012; Leslie et al. 2012, 2018; Uckele et al. 2021). During this time, major mountain systems worldwide—such as the Himalayas, the Rocky Mountains, and the Qinghai–Tibet Plateau (QTP)—attained their current heights and topographies. This geological upheaval, combined with a shift towards cooler and more arid conditions (Graham 1999), likely encouraged speciation processes by creating both geographic and ecological barriers to gene flow (Leslie et al. 2012; Mao et al. 2010).

Junipers exhibit notable diversification patterns associated with the Miocene, especially in clades such as those found in the QTP and the Americas. However, although the Miocene has been hypothesized as a critical time for conifer diversity, there is no evidence of increased net diversification rates for junipers during this period (Leslie et al. 2018; Sundaram et al. 2019). Despite prior expectations (Mao et al. 2010; Uckele et al. 2021), our analyses did not detect an increase in net diversification among global juniper species in the Miocene. Instead, our findings suggest a deceleration in the evolutionary rate of at least one climate‐related trait: precipitation in the driest quarter (Bio17) (Figure 1). This deceleration aligns with the evolutionary trajectory of the turbinate‐cone clade in the *Sabina* section, primarily found on the QTP, which experiences some of the most arid and coldest conditions in the genus (Adams 2014; Adams and Schwarzbach 2012).

Our analyses using Bayou reveal the critical role that climatic variables play in the evolution of juniper species on the QTP (Figure 2). For instance, key precipitation and temperature parameters—such as minimum temperatures for both the driest quarter and coldest month—fit well with an Ornstein–Uhlenbeck (OU) evolutionary model showing two distinct optimal values (*θ*) or climatic *optima*. This model suggests that juniper lineages have experienced a major climatic divergence in their evolutionary histories, with Qinghai–Tibet species adapted to colder conditions (Figure 2b–d). These trends may reflect the geological history of the QTP and the Himalayas, which have altered regional climates, primarily through moisture loss and the establishment of the Asian Monsoon system (Royden, Clark Burchfiel, and Van Der Hilst 2008; Su et al. 2019). According to proposed phylogenies (Mao et al. 2010), turbinate‐seed juniper species likely originated between 23 and 14 million years ago, coinciding with intense tectonic activity and uplifts in the QTP. This geological activity has contributed significantly to the region's biodiversity (Favre et al. 2015; Spicer 2017).

### 4.2 General Patterns of Phylogenetic Niche Conservation

Phylogenetic niche conservatism is said to be common among conifers, indicating that climatic adaptation and ecological divergence may have had a limited role in the broader diversification history of these species (Cruz‐Nicolás et al. 2021, 2023; Jin et al. 2021; Sundaram and Leslie 2021). However, climatic differentiation is evident among more recently diverged conifer species, as seen in the diversification during the Pleistocene (Martínez de León, Castellanos‐Morales, and Moreno‐Letelier 2022; Moreno‐Letelier, Mastretta‐Yanes, and Barraclough 2014; Ortíz‐Medrano et al. 2016). The Bayou results for precipitation variables support that the impact of climate on juniper diversification is most pronounced at deeper evolutionary timescales, such as the Miocene, suggesting adaptive responses to long‐term environmental changes driven by orogenic events. Meanwhile, more recent niche differentiation within species appears to be associated with complex dynamics, such as those arising from Pleistocene climatic cycles.

In terms of macroevolutionary patterns, our results support a trend of phylogenetic niche conservatism (PNC) within junipers, with climatic niches remaining consistent within lineages. In particular, while the QTP juniper species exhibit distinct climatic components relative to other junipers, these Tibetan species display a much conserved climatic niche. The maintenance of this temperature‐related niche among Tibetan species may stem from phylogenetic constraints on climatic niche evolution, which often lead to a general pattern of “soft allopatry” in scenarios of geological heterogeneity and climatic shifts, such as those experienced in Miocene eastern Asia (Pyron et al. 2015). In soft allopatry, a broadly shared climatic niche splits into environmental extremes on either side of a weak geographic barrier, fostering geographic isolation and seemingly divergent but fundamentally similar climatic niches (Fisher‐Reid, Kozak, and Wiens 2012; Pyron et al. 2015; Wiens and Graham 2005). Within eastern Asian junipers, the genetic diversity patterns observed are consistent with allopatric speciation and some gene flow between Tibetan species (Li et al. 2011; Zhang et al. 2005).

### 4.3 Centers of Diversity

Our results highlight the importance of considering each center of diversity on its own (Figures 3 and 4). Both in the linear models and the CANAPE analysis, each region had quite different results regarding the major factors explaining variance in the calculated net diversification. Briefly, our results are consistent with the reported diversity hotspots for juniper species (Adams 2014; Farjon 2003; Sundaram et al. 2019). Moreover, our results support the idea of each center of diversity as a unique combination of variables and the lack of a common *driver* of diversification for all diversity hotspot proposed by Sundaram et al. (2019, 2021). Here, the final models selected suggest that climatic niche has had a low effect on net diversification for the three studied areas (Figure 3). In the CANAPE analysis, we found the existence of 4 phylogenetically endemic regions: the Antilles, Central China, southern Mexico, and the Mediterranean Basin (Figure 4a–c).

### 4.4 Central China

Our results highlight the relevance of topographic conditions across the QTP in shaping diversity in juniper species (Sundaram et al. 2019; Wen et al. 2014). We found that endemism results correspond to a major increase in neo‐endemism specifically in this region. Combined, our results in niche evolution and diversification seem to stress the relevance of tectonic activity in the formation of the QTP during the Miocene for juniper diversity. According to the high number of neo‐endemism, diversification due to allopatry was rather rapid. Later, the evolution of climate tolerances to new conditions could explain patterns in niche evolution. Our results suggest that the region is highly attractive for the study of climate adaptation (Spicer 2017; Su et al. 2019; Wang et al. 2008).

### 4.5 The Mediterranean Basin

We found no significant climate shifts in niche evolution associated with species inhabiting the Mediterranean Basin. Additionally, the most relevant variables associated with diversification rates were species richness and phylogenetic endemism (Figure 3c). This means that diversification rates are high in places with a low number of species and species that are highly endemic. This result is reasonable and consistent with our findings in endemic regions. We found a relatively low but significant level for paleoendemic regions. Considered collectively, these results suggest that the main process that shape diversity are the accumulation of species probably due to migration and colonization from very distant species. Accordingly, we might add in this regard that the oldest species are found in this region, and therefore intensive extinction events might have occurred, giving this region a high number of distant species (Mao et al. 2010; Sundaram et al. 2019).

### 4.6 The Americas

Surprisingly, morphological evolution showed an elevated level of importance in explaining the net diversification results in the American continent (Figure 3d). This could be explained by the coexistence of three different *Juniperus* lineages that arrived independently in North America and even had different proposed dispersion routes which could account for more diversity in the number of seeds per cone (Adams 2000, 2014; Mao et al. 2010). Therefore, at least in this area, morphological evolution is somewhat related to net diversification. The standardized coefficients suggest that in this region the number of seeds per cone might have evolved according to speciation events. The opposite can be said about habit, which has a negative relation to diversification and is quite conserved.

In conclusion, our results highlight the importance of testing several potential drivers of speciation when studying diversification rates in taxa with broad distribution. The biogeographic history of *Juniperus* is complex and has been marked by multiple continental colonizations (Mao et al. 2010). However, the temporal concordance between the diversifications of junipers of the QTP and North America suggested a common cause, but our results show that one was climate‐driven, and the other is linked to the morphological evolution of seed cones. Juniper seeds are animal‐dispersed; therefore, it is still important to analyze our results in relation to potential dispersers, which might also influence speciation rates in the high‐diversity areas of North America.

The study of climatic divergence among lineages relies deeply on a good understanding of species diversity in phylogenies and a strong characterization of climatic niches in the geographic space at least. Evidence suggests that juniper species hide more cryptic diversity than thought, especially in North America (e.g., Martínez de León, Castellanos‐Morales, and Moreno‐Letelier 2022; Moreno‐Letelier, Mastretta‐Yanes, and Barraclough 2014). Future research should address this possibility in the light of concordance of evolutionary dynamics and climatic characterizations.

## Author Contributions

**Rodrigo Martínez de León:** conceptualization (supporting), data curation (lead), formal analysis (lead), investigation (equal), methodology (equal), writing – original draft (equal), writing – review and editing (equal). **Alejandra Moreno‐Letelier:** conceptualization (lead), data curation (supporting), formal analysis (equal), funding acquisition (lead), investigation (equal), methodology (equal), project administration (lead), supervision (lead), writing – original draft (equal), writing – review and editing (equal).

## Conflicts of Interest

The authors declare no conflicts of interest.

## Supporting information


Data S1

## Data Availability

R scripts and data frames necessary to replicate the analyses above are available at Figshare.com (https://doi.org/10.6084/m9.figshare.26156182).
