# Supplementary material for: Spatiotemporal Diversification of Global Junipers: Traces of Niche Conservatism and Trait‐Dependent Diversification
Source: Ecol Evol. 2025 Feb 12;15(2):e70910. doi: 10.1002/ece3.70910 (PMC11815337; doi:10.1002/ece3.70910)
Supplement: Supplementary file 1 — Data S1 [file ECE3-15-e70910-s001.docx]

**Spatiotemporal diversification of global junipers: Traces of niche conservatism and trait-dependent diversification.**

Rodrigo Martínez de León1,2

Alejandra Moreno-Letelier2*

1 Posgrado en Ciencias Biológicas, Universidad Nacional Autónoma de México, Coyoacán, 04510 Ciudad de México, México

2 Jardín Botánico, Instituto de Biología, Universidad Nacional Autónoma de México, Coyoacán, 04510 Ciudad de México, México

**Supplementary Materials.**

**S1. Data collection.**

**Table S1.1. Species and number of records. For each species the number of total records is shown after filters and thinning were applied.**

| Species | Total number of records |
| --- | --- |
| *Juniperus angosturana* R.P. Adams | 61 |
| *Juniperus ashei* J. Buchholz | 499 |
| *Juniperus barbadensis* L. | 18 |
| *Juniperus bermudiana* L. | 11 |
| *Juniperus blancoi* Martínez | 7 |
| *Juniperus californica* Carrière | 662 |
| *Juniperus chinensis* L. | 247 |
| *Juniperus coahuilensis* (Martínez) Gaussen ex R.P. Adams | 93 |
| *Juniperus comitana* Martínez | 44 |
| *Juniperus communis* L. | 1103 |
| *Juniperus convallium* Rehder & E.H. Wilson | 53 |
| *Juniperus deltoides* R.P. Adams | 247 |
| *Juniperus deppeana* Steud. | 872 |
| *Juniperus drupacea* Labill. | 48 |
| *Juniperus durangensis* Martínez | 75 |
| *Juniperus excelsa* Pursh | 173 |
| *Juniperus flaccida* Schltdl. | 463 |
| *Juniperus formosana* Hayata | 254 |
| *Juniperus gamboana* Martínez | 47 |
| *Juniperus gracilior*  Pilg. | 18 |
| *Juniperus horizontalis*  Moench. | 880 |
| *Juniperus indica* Bertol. | 115 |
| *Juniperus komarovii* Florin | 16 |
| *Juniperus monosperma* (Engelm.) Sarg. | 601 |
| *Juniperus monticola* Martínez | 127 |
| *Juniperus occidentalis*  Hook. | 760 |
| *Juniperus osteosperma* (Torr.) Little | 1068 |
| *Juniperus oxycedrus* Spach | 417 |
| *Juniperus phoenicea*  L. | 611 |
| *Juniperus pinchotii* Sudw. | 293 |
| *Juniperus pingii* W.C. Cheng ex Ferré | 73 |
| *Juniperus polycarpos* K. Koch | 26 |
| *Juniperus procera* Hochst. ex Endl. | 161 |
| *Juniperus procumbens* (Siebold ex Endl.) Siebold ex Miq. | 20 |
| *Juniperus przewalskii* Kom. | 29 |
| *Juniperus pseudosabina*  Fisch. & C.A. Mey. | 488 |
| *Juniperus rigida* Siebold & Zucc. | 539 |
| *Juniperus sabina* Spach | 238 |
| *Juniperus saltillensis* M.T. Hall | 52 |
| *Juniperus saltuaria* Rehder & E.H. Wilson | 75 |
| *Juniperus saxicola* Britton & P. Wilson | 6 |
| *Juniperus scopulorum* Sarg. | 446 |
| *Juniperus semiglobosa* Regel | 186 |
| *Juniperus squamata* Lamb. | 165 |
| *Juniperus taxifolia* Hook. & Arn. | 26 |
| *Juniperus thurifera* L. | 269 |
| *Juniperus tibetica* Kom. | 95 |
| *Juniperus virginiana* L. | 669 |


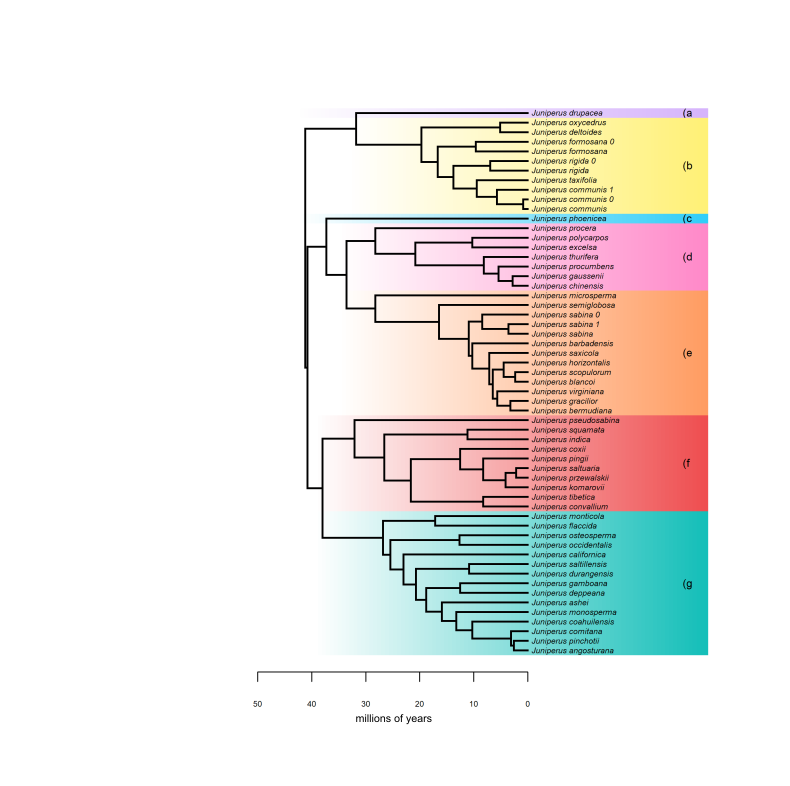


**Figure S1.1 Recovered molecular phylogenetic relationships within *Juniperus*. Principal recovered clades by Mao t al (2010). a) Section *Caryocedrus.* b) Section *Juniperus*. c) Section *Sabina* Clade V. d) Section *Sabina* Clade IV (Smooth leave juniper clade). e) Section *Sabina* Clade III. f) Section *Sabina* Clade I (East Asian Juniper clade). g) Section *Sabina* Clade II (North American marginated clade).**

**Table S1.2. Bayou’s results for variable and the estimated effective sample size for 20 million iterations. The number of estimated shifts in climate regime (i.e. K) with posterior above 0.8 is presented.**

| Variable | Number of K (shifts) | ESS |
| --- | --- | --- |
| Bio03 | 1 | 600 |
| Bio06 | 1 | 800 |
| Bio09 | 1 | 800 |
| Bio10 | 2 | 300 |
| Bio17 | 4 | 800 |
| Bio19 | 4 | 800 |
| Bio02 | 0 | 1500 |
| Bio07 | 0 | 1000 |
| Bio08 | 0 | 1000 |
| Bio15 | 0 | 1200 |
| Bio16 | 0 | 1000 |
| Bio18 | 0 | 1500 |

**S2. Correlation matrix for climatic variables.**

**Table 2.1 Pearson’s correlation r for each climatic variable used**.

**S3. Standardised coefficients of spatial and regular models.**

**Table S3.1 Standardised coefficients and likelihood values for the OSL model for the Norh American region.**

|  | Estimate | Std. Error | t value | Pr(>\|t\|) |
| --- | --- | --- | --- | --- |
| (Intercept) | 1.405e-14 | 3.291e-02 | 0.000 | 1.000000 |
| bio 10 mean | -3.079e-01 | 6.603e-02 | -4.664 | **6.31e-06 ***** |
| Bio 12 mean | -1.060e-01 | 6.052e-02 | -1.751 | 0.081790 |
| Bio 15 mean | 3.154e-01 | 5.185e-02 | 6.083 | **7.73e-09 ***** |
| Bio 8 mean | -1.503e-01 | 6.012e-02 | -2.501 | **0.013352 *** |
| Elevation mean | -2.052e-01 | 8.584e-02 | -2.391 | **0.017932 *** |
| Elevation sd | 2.488e-01 | 6.862e-02 | 3.625 | **0.000383 ***** |
| beta17 | 7.942e-02 | 8.352e-02 | 0.951 | 0.343052 |
| Phylogenetic diversity | -1.533e-01 | 5.354e-02 | -2.863 | **0.004734 **** |
| Phylogenetic endemism | -2.353e-01 | 6.018e-02 | -3.909 | **0.000134 ***** |
| Proportion of transition of habit | -7.881e-01 | 7.362e-02 | -10.704 | **< 2e-16 ***** |
| Species richness | 1.429e-01 | 5.002e-02 | 2.856 | **0.004828 **** |
| Proportion of transition of seed number | 7.736e-01 | 1.070e-01 | 7.234 | **1.59e-11 ***** |

Signif. codes: ***= 0.001; **= 0.01; *= 0.05
Residual standard error: 0.4428 on 168 df
**Multiple R^2^: 0.817**
Adjusted R^2^: 0.8039
F-statistic: 62.5 on 12 and 168 DF
p-value: < 2.2e-16

**Table S3.2 Standardised coefficients and likelihood values for the Spatial Error model for the Norh American region.**

|  | Estimate | Std. Error | z value | Pr(>\|z\|) |
| --- | --- | --- | --- | --- |
| (Intercept) | -0.012622 | 0.042726 | -0.2954 | 0.7676690 |
| Bio 10 mean | -0.255896 | 0.065107 | -3.9304 | 8.480e-05 |
| Bio 12 mean | -0.130884 | 0.056074 | -2.3341 | 0.0195890 |
| Bio 15 mean | 0.305419 | 0.055558 | 5.4973 | 3.857e-08 |
| Bio 8 mean | -0.182139 | 0.063251 | -2.8796 | 0.0039813 |
| Elevation mean | -0.208166 | 0.084915 | -2.4515 | 0.0142279 |
| Elevation sd | 0.256124 | 0.071188 | 3.5978 | 0.0003209 |
| beta17 | 0.075629 | 0.088725 | 0.8524 | 0.3939963 |
| Phylogenetic diversity | -0.154417 | 0.053118 | -2.9071 | 0.0036483 |
| Phylogenetic endemism | -0.230014 | 0.059083 | -3.8931 | 9.898e-05 |
| Proportion of transition of habit | -0.810837 | 0.071544 | -11.3334 | < 2.2e-16 |
| Species richness | 0.156530 | 0.045530 | 3.4379 | 0.0005862 |
| Proportion of transition of seed number | 0.807853 | 0.109322 | 7.3897 | 1.472e-13 |

Lambda: 0.33963
LR test value: 31.328
p-value: 2.1794e-08
Asymptotic standard error: 0.051962
 z-value: 6.5361, p-value: 6.3155e-11
Wald statistic: 42.72, p-value: 6.3155e-11

Log likelihood: -86.97137 for error model
ML residual variance (squared): 0.14324, (sigma: 0.37847)
**Nagelkerke pseudo-R^2^: 0.84608**Number of observations: 181
Number of parameters estimated: 15

**Table S3.3 Standardised coefficients and likelihood values for the OSL model for Europe region.**

|  | Estimate | Std. Error | t value | Pr(>\|t\|) |
| --- | --- | --- | --- | --- |
| (Intercept) | -1.131e-14 | 4.815e-02 | 0.000 | 1.00000 |
| beta6 | 1.931e-01 | 6.326e-02 | 3.053 | **0.00310 **** |
| Bio 4 mean | 5.161e-01 | 6.649e-02 | 7.762 | **2.72e-11 ***** |
| Bio 15 mean | 2.056e-02 | 7.085e-02 | 0.290 | 0.77241 |
| Bio 9 mean | 8.603e-02 | 8.009e-02 | 1.074 | 0.28608 |
| Bio 19 mean | 2.425e-01 | 5.765e-02 | 4.206 | **6.88e-05 ***** |
| Elevation mean | -2.139e-01 | 5.869e-02 | -3.645 | **0.00048 ***** |
| Phylogenetic diversity | 7.674e-01 | 1.106e-01 | 6.941 | **1.02e-09 ***** |
| Phylogenetic endemism | 4.348e-01 | 6.313e-02 | 6.888 | **1.29e-09 ***** |
| Species richness | -8.170e-01 | 1.225e-01 | -6.669 | **3.33e-09 ***** |
| Proportion of transition of seed number | 6.074e-01 | 8.170e-02 | 7.435 | **1.16e-10 ***** |

Signif. codes: ***= 0.001; **= 0.01; *= 0.05
Residual standard error: 0.4543 on 78 degrees of freedom
**Multiple R-squared: 0.8171**Adjusted R-squared: 0.7936
F-statistic: 34.84 on 10 and 78 DF; p-value: < 2.2e-16

**Table S3.4 Standardised coefficients and likelihood values for the OSL model for the Asian region.**

|  | Estimate | Std. Error | t value | Pr(>\|t\|) |
| --- | --- | --- | --- | --- |
| (Intercept) | 1.044e-15 | 5.407e-02 | 0.000 | 1.000000 |
| Bio 1 mean | 5.119e-01 | 1.326e-01 | 3.860 | **0.000213 ***** |
| Bio 13 mean | 3.366e-02 | 1.063e-01 | 0.317 | 0.752244 |
| Bio 9 mean | -4.359e-01 | 1.215e-01 | -3.589 | **0.000540 ***** |
| Bio 2 mean | -1.185e-01 | 1.105e-01 | -1.072 | 0.286492 |
| Bio 4 mean | -1.094e-01 | 8.136e-02 | -1.345 | 0.182131 |
| Elevation sd | -1.373e-01 | 8.146e-02 | -1.686 | 0.095311 |
| Species richness | -2.438e-01 | 1.169e-01 | -2.086 | **0.039821 *** |
| Phylogenetic diversity | 2.986e-01 | 1.080e-01 | 2.764 | **0.006918 **** |
| Phylogenetic endemism | -2.892e-01 | 1.044e-01 | -2.769 | **0.006823 **** |
| beta19 | -7.316e-01 | 1.006e-01 | -7.274 | **1.24e-10 ***** |

Signif. codes: ***= 0.001; **= 0.01; *= 0.05

Residual standard error: 0.5434 on 90 degrees of freedom
**Multiple R-squared: 0.7342**
Adjusted R-squared: 0.7047
F-statistic: 24.87 on 10 and 90 DF, p-value: < 2.2e-16

**Table S3.5 Standardised coefficients and likelihood values for the Spatial Error model for the Asian region.**

|  | Estimate | Std. Error | z value | Pr(>\|z\|) |
| --- | --- | --- | --- | --- |
| (Intercept) | -0.0019209 | 0.0644599 | -0.0298 | 0.9762271 |
| Bio 1 mean | 0.4816084 | 0.1312048 | 3.6707 | 0.0002419 |
| Bio 13 mean | 0.0145367 | 0.1080467 | 0.1345 | 0.8929751 |
| Bio 9 mean | -0.3701073 | 0.1176547 | -3.1457 | 0.0016569 |
| Bio 2 mean | -0.0836819 | 0.1061501 | -0.7883 | 0.4305003 |
| Bio 4 mean | -0.1457275 | 0.0878669 | -1.6585 | 0.0972161 |
| Elevation sd | -0.1536144 | 0.0837809 | -1.8335 | 0.0667245 |
| Species richness | -0.2884255 | 0.1108213 | -2.6026 | 0.0092515 |
| Phylogenetic diversity | 0.3475316 | 0.0951207 | 3.6536 | 0.0002586 |
| Phylogenetic endemism | -0.3223763 | 0.1004386 | -3.2097 | 0.0013288 |
| beta19 | -0.7654976 | 0.1027797 | -7.4479 | 9.481e-14 |


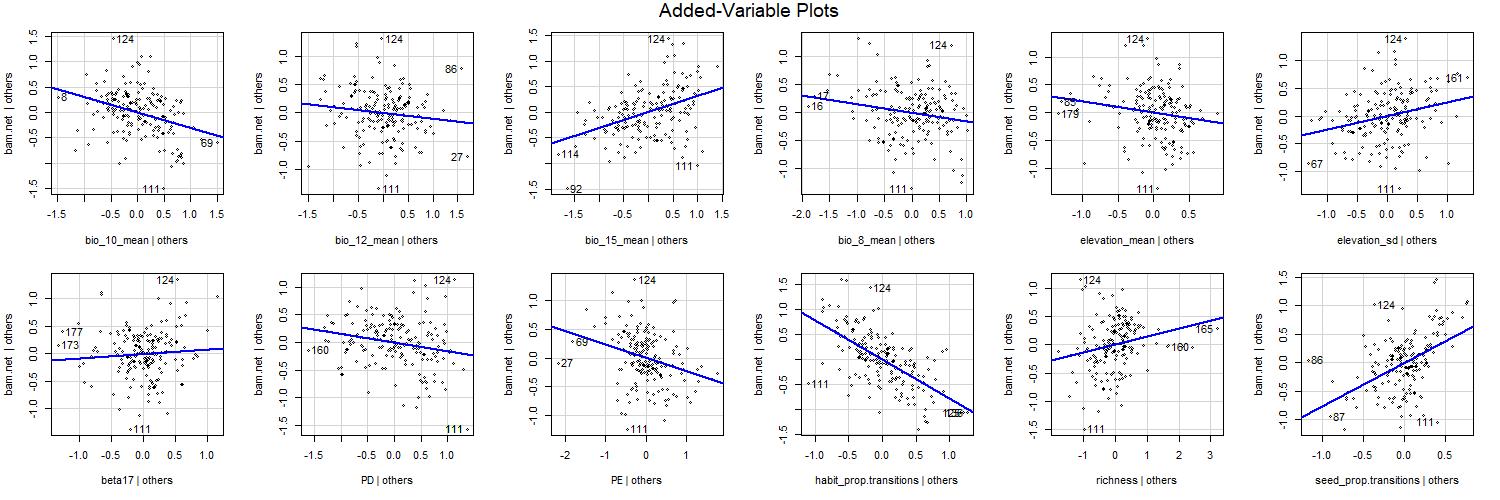
**S4. Partial residuals for OLS models.**


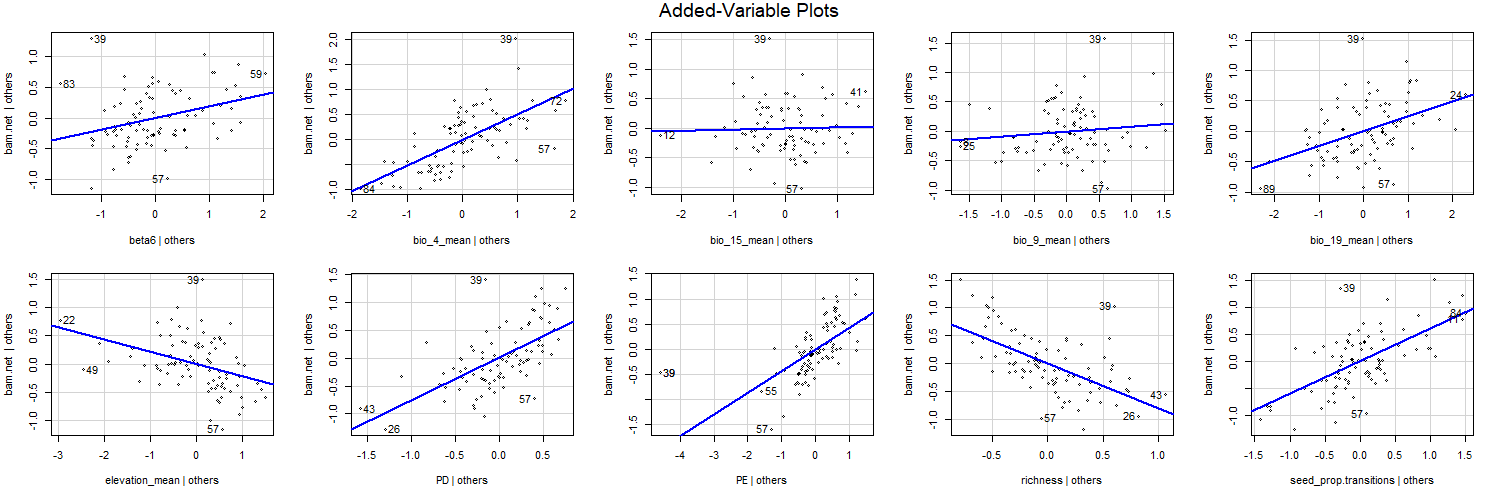
Fig S4.1. Partial residual plot for the OLS model in the North American region. Diversification values are shown in response to residuals of a regression model fitted without the interest predictor.

Fig S4.2. Partial residual plot for the OLS model in the Europe region. Diversification values are shown in response to residuals of a regression model fitted without the interest predictor.


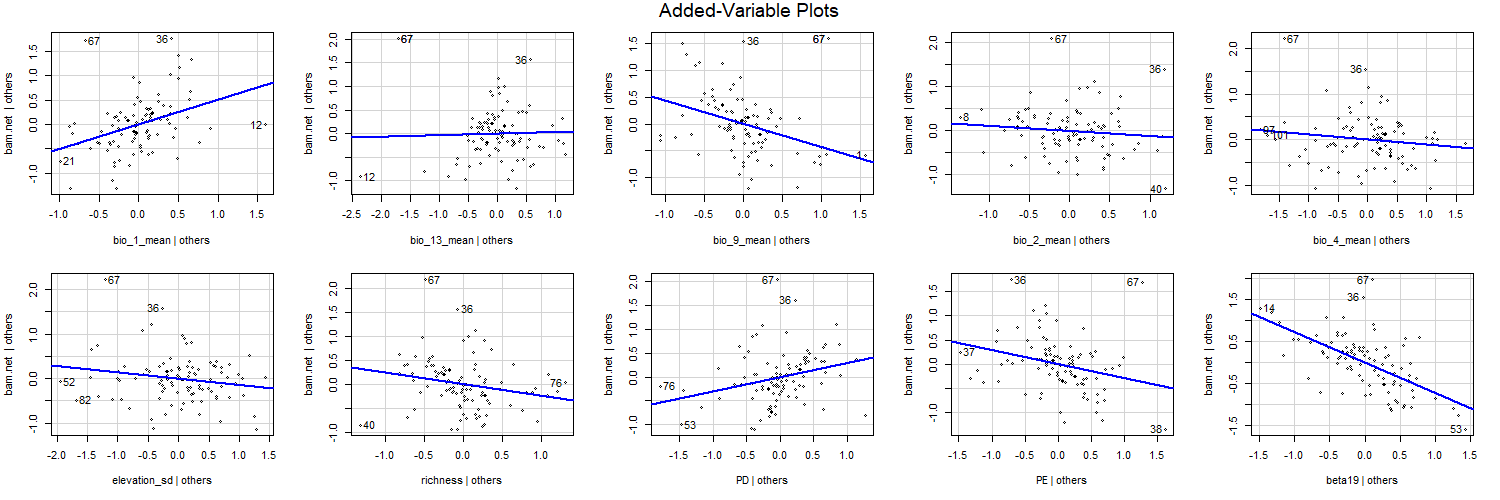
Fig S4.3. Partial residual plot for the OLS model in the Asian region. Diversification values are shown in response to residuals of a regression model fitted without the interest predictor.

**S5. Residuals for OLS models**

We present the resulting plots for the residual vs fitted values for each OLS model used.

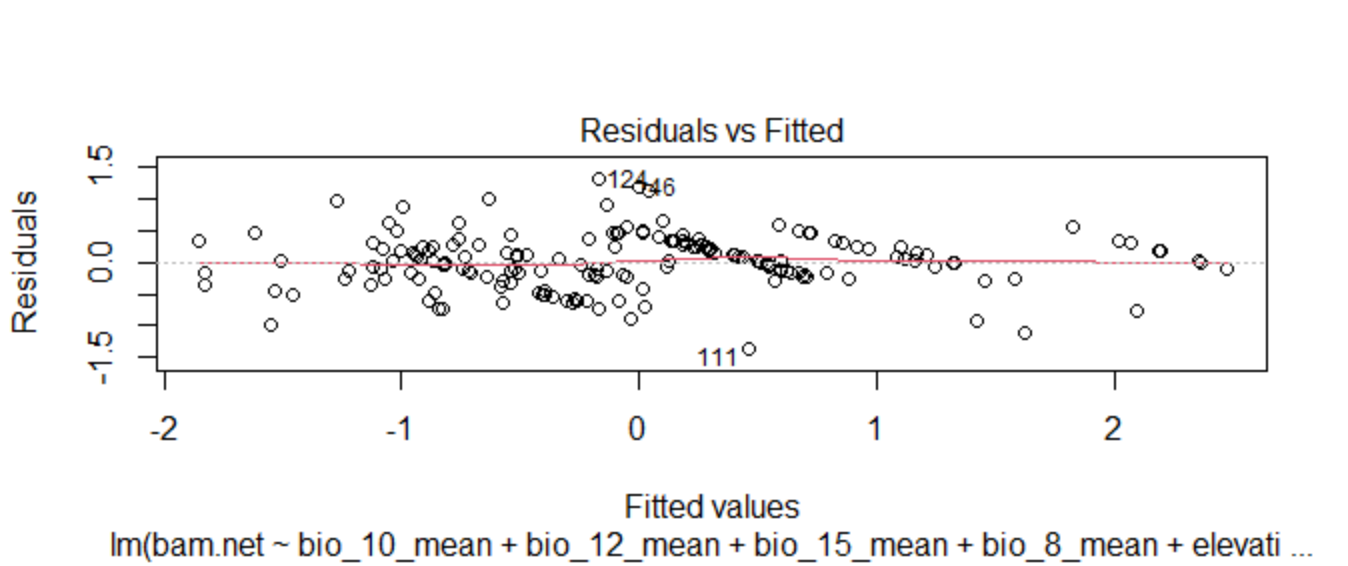


Fig S5.1. North America OLS model residuals versus fitted values.


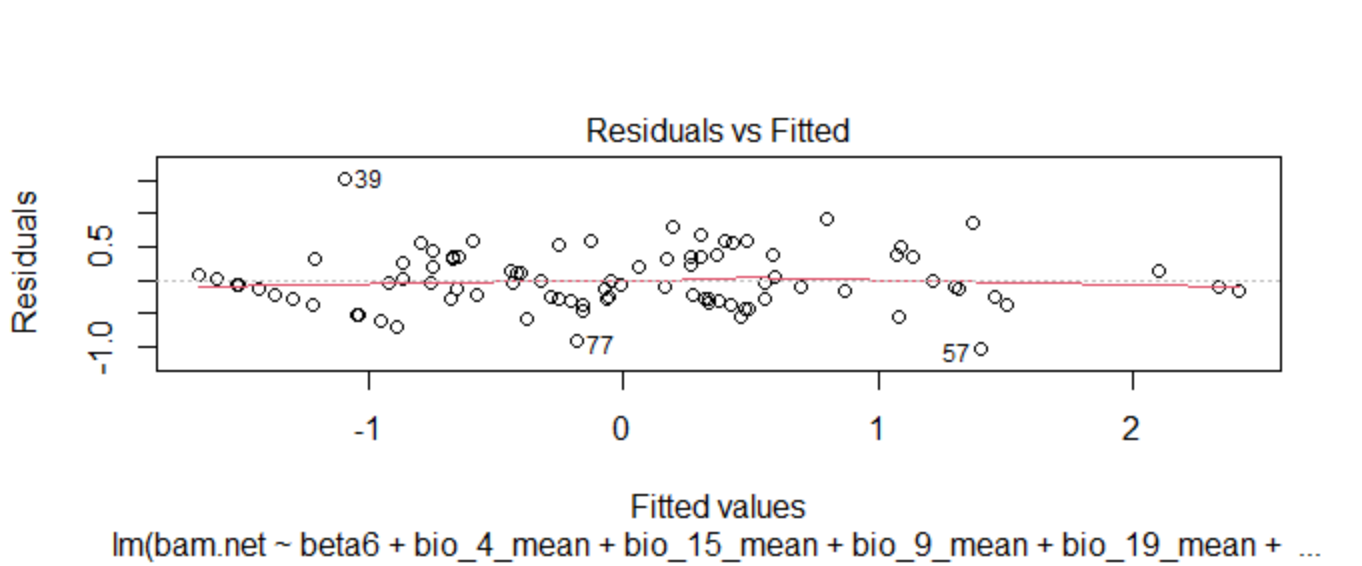


Fig S5.2. Europe OLS model residuals versus fitted values.


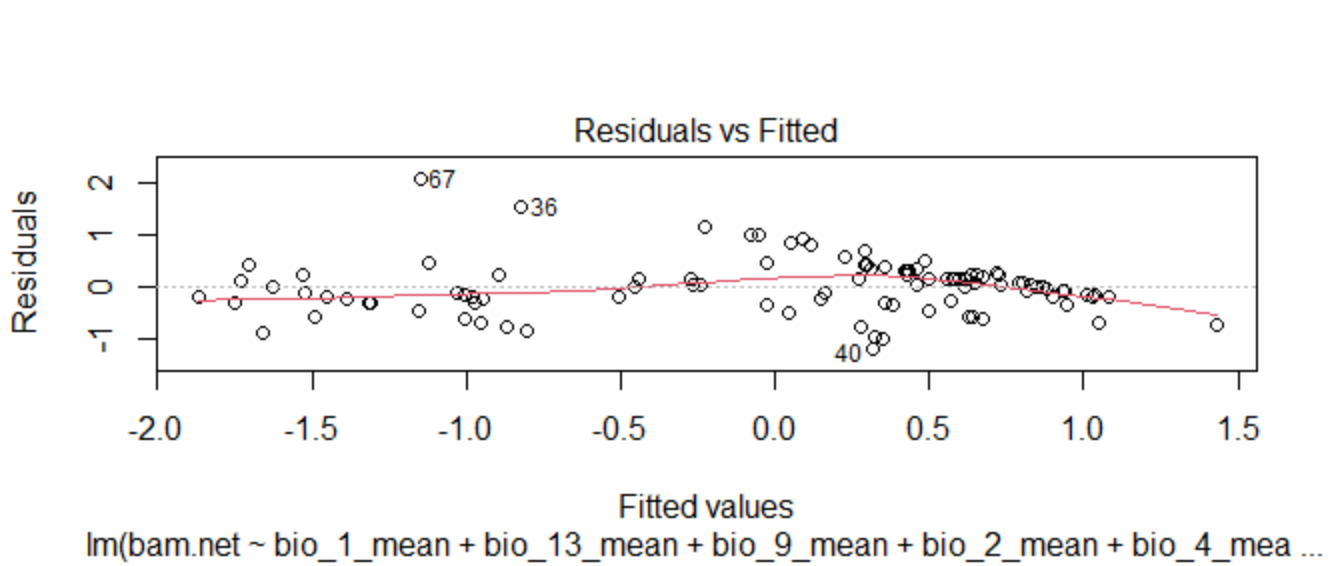


Fig S5.3. Asia OLS model residuals versus fitted values.

**S6. Predictor’s individual contribution to overall variance explained by OLS models.**

**Table S6.1 Contribution of individual predictors to overall variance in the North American region OLS model.
These values correspond to mean contribution based on 1000 bootstrapping.**

| Predictor | Variance explained (proportion) |
| --- | --- |
| Bio 10 mean | 0.1432 |
| Bio 12 mean | 0.0243 |
| Bio 15 mean | 0.0237 |
| Bio 8 mean | 0.0413 |
| Beta 17 | 0.0164 |
| Elevation mean | 0.0265 |
| Elevation sd | 0.1394 |
| Phylogenetic diversity | 0.0920 |
| Phylogenetic endemism | 0.02176 |
| Proportion of habit transitions | 0.13940 |
| Species richness | 0.01903 |
| Proportion of number of seed transitions | 0.12822 |
| Unexplained | 0.18466 |

**Table S6.2 Contribution of individual predictors to overall variance in the Europe region OLS model.
These values correspond to mean contribution based on 1000 bootstrapping.**

| Predictor | Variance explained (proportion) |
| --- | --- |
| Beta 6 | 0.013266 |
| Bio 4 mean | 0.250538 |
| Bio 15 mean | 0.022970 |
| Bio 9 mean | 0.043606 |
| Bio 19 mean | 0.049511 |
| Elevation mean | 0.023710 |
| Phylogenetic diversity | 0.073760 |
| Phylogenetic endemism | 0.068400 |
| Species richness | 0.053813 |
| Proportion of number of seeds transitions | 0.217405 |
| Unexplained | 0.183021 |

**Table S6.3 Contribution of individual predictors to overall variance in the Asian region OLS model.
These values correspond to mean contribution based on 1000 bootstrapping.**

| Predictor | Variance explained (proportion) |
| --- | --- |
| Bio 1 mean | 0.083740 |
| Bio13 mean | 0.046874 |
| Bio 9 mean | 0.058180 |
| Bio 2 mean | 0.077579 |
| Bio 4 mean | 0.008363 |
| Elevation sd | 0.143758 |
| Species richness | 0.016080 |
| Phylogenetic diversity | 0.027990 |
| Phylogenetic endemism | 0.039390 |
| Beta 19 | 0.232265 |
| Unexplained | 0.265781 |
